# Trefoil Factor Family (TFF) Peptides and Their Links to Inflammation: A Re-evaluation and New Medical Perspectives

**DOI:** 10.3390/ijms22094909

**Published:** 2021-05-06

**Authors:** Werner Hoffmann

**Affiliations:** Institute of Molecular Biology and Medicinal Chemistry, Otto-von-Guericke University Magdeburg, Leipziger Str. 44, 39120 Magdeburg, Germany; werner.hoffmann@med.ovgu.de

**Keywords:** gastric cancer, reactive oxygen species, inflammation, innate immunity, macrophages, trefoil factor, lectin, FCGBP, mucin, receptor blocking

## Abstract

Trefoil factor family peptides (TFF1, TFF2, TFF3), together with mucins, are typical exocrine products of mucous epithelia. Here, they act as a gastric tumor suppressor (TFF1) or they play different roles in mucosal innate immune defense (TFF2, TFF3). Minute amounts are also secreted as endocrine, e.g., by the immune and central nervous systems. As a hallmark, TFF peptides have different lectin activities, best characterized for TFF2, but also TFF1. Pathologically, ectopic expression occurs during inflammation and in various tumors. In this review, the role of TFF peptides during inflammation is discussed on two levels. On the one hand, the expression of TFF1-3 is regulated by inflammatory signals in different ways (upstream links). On the other hand, TFF peptides influence inflammatory processes (downstream links). The latter are recognized best in various *Tff*-deficient mice, which have completely different phenotypes. In particular, TFF2 is secreted by myeloid cells (e.g., macrophages) and lymphocytes (e.g., memory T cells), where it modulates immune reactions triggering inflammation. As a new concept, in addition to lectin-triggered activation, a hypothetical lectin-triggered inhibition of glycosylated transmembrane receptors by TFF peptides is discussed. Thus, TFFs are promising players in the field of glycoimmunology, such as galectins and C-type lectins.

## 1. Introduction

### 1.1. TFF Peptides: The “Classical” View

**Figure 1:**
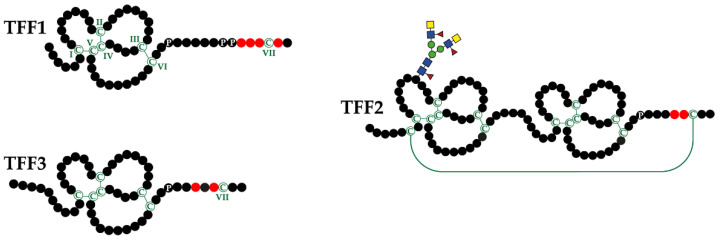
Schematic structures of the three human TFF peptides. Cysteine residues (C; numbering in Roman numerals) and disulfide bridges are shown in green. TFF2 contains an additional disulfide bridge between Cys-6 and Cys-104 creating a circular structure; also represented are the proline residues (P) at the C-terminal outside the TFF domains. Acid residues in proximity to the C-terminal cysteine residues that modify its reactivity (change of pKa) are shown in red.

In humans, secretory trefoil factor family (TFF) peptides comprise TFF1, TFF2, and TFF3 (reviews: [1,2,3,4]). They share characteristic cysteine-rich modules (TFF domains [5]; formerly trefoil domains [6], P-domains [7]), where six cysteine residues form three intramolecular disulfide bridges in the order Cys^I-V^, Cys^II-IV^, and Cys^III-VI^ (Figure 1). Both TFF1 and TFF3 contain a single TFF domain and a 7^th^ cysteine residue at their C-terminal outside the TFF domain (Cys^VII^). In contrast, TFF2 contains two TFF domains and two additional cysteine residues, the latter connecting the C- and N-terminal via a disulfide bridge (Figure 1). There are indications that the resulting circular structure occurs in different forms (maybe supercoils) [8,9]. In spite of their overall similarity, there is probably a major structural difference between TFF1 and TFF3 concerning the nucleophilicity of Cys^VII^, which is enhanced in TFF1 by steric exposure (neighboring proline residues, Figure 1). Remarkably, human TFF2 is N-glycosylated (gastric TFF2 bears an unusual fucosylated LacdiNAc oligosaccharide [10]); whereas murine and porcine TFF2 lack N-glycosylation sites. Generally, TFF peptides have been characterized from frogs to humans thus far [11].

The major amounts of TFF peptides are secreted from mucous epithelia, where they are released together with mucins in an exocrine manner [4,11,12,13]. TFF1 is mainly expressed in gastric surface mucous cells (together with the mucin MUC5AC), TFF2 is—together with MUC6—restricted to gastric mucous neck cells, antral gland cells and duodenal Brunner’s glands, whereas TFF3 is a typical product of intestinal goblet cells and most other mucous epithelia and their glands. Consequently, TFFs are constituents of mucus barriers and appear also in the corresponding body fluids, such as saliva, gastric juice, and urine, as well as in tears and breast milk [3].

Furthermore, minute amounts of TFF peptides undergo endocrine secretion. Typical examples are lymphoid organs and tissues (thymus, bone marrow, spleen, lymph nodes, gut-associated lymphatic tissue, etc. [14,15,16,17,18]), the brain, the thyroid, and the pancreas [4]. That is the reason why TFFs are also detectable in normal human serum [3,4].

In the past, the biological/molecular function of TFF peptides was explained as a paradigm of their migratory effects, which were postulated to stimulate the rapid repair of mucous epithelia by a process called “restitution” [19]. Subsequently, many publications appeared reporting motogenic effects in vitro and protective or healing effects of TFFs in vivo (compilation: [11]). The three TFF peptides showed remarkably similar activities. Taken together, the effects observed were not really convincing as they were hardly detectable in vitro and occurred at concentrations of 10^−6^ to 10^−7^ M or even above [4,12,20]. This concentration is atypical of classical receptor/peptide ligand interactions and is in agreement with a failure to detect high-affinity TFF binding proteins [21]. Thus, rather low-affinity binding can be expected, which would be in agreement with the known, but different, lectin activities of TFF peptides [4,22]. Such a hypothetical function of TFF peptides as activating lectin ligands for a plethora of transmembrane glycoproteins triggering signal transduction processes has already been proposed in the past [23]. Currently, the following transmembrane proteins were reported to have a binding affinity for TFF peptides: β1 integrin [24], CRP-ductin/DMBT1^gp340^ [24,25], CXCR4 and CXCR7 [26,27,28], PAR2 [29], PAR4 [30], LINGO2 [31], and LINGO3 [32]. Remarkably, many of these transmembrane proteins are known to support cell migration processes. Based on this rather diverse list, one might also expect that more members will be added in the near future (e.g., transmembrane mucins and other Cluster of Differentiation/CD molecules). However, it is the challenge now to clarify unambiguously whether signal transduction processes are triggered specifically by TFF peptides and to characterize the potential ligand binding in detail (e.g., dose-response curves for different forms of TFF1, TFF2, and TFF3), a major question being whether TFF peptide binding occurs via lectin or protein–protein interactions. Finally, the question arises on the biological significance of such processes in mucous epithelia (exocrine secretion) or mainly in organs with endocrine secretion of minute amounts of TFF peptides.

However, the concentrations of TFF peptides in mucous epithelia are rather high and it is unlikely that under physiological conditions they mainly act as activating high-affinity ligands of transmembrane receptors triggering intracellular signaling processes. This view is strengthened by long lasting systematic studies concerning the natural forms of TFF peptides in mucous epithelia [33,34,35,36,37]. As the major result, surprisingly, TFF peptides were found to appear in different molecular forms indicating diverse molecular functions. This led to a change in the paradigm concerning their molecular functions in healthy mucous epithelia [4].

### 1.2. Exocrine TFF Peptides Occur in Different Molecular Forms and Have Diverse Molecular Functions

Gastric TFF1 occurs mainly as a monomer with a highly exposed free thiol group at Cys^VII^ as shown for humans [37], mice [9], and the *Xenopus laevis* ortholog xP1 [38]. Such an unpaired cysteine residue is unusual for secretory proteins, which normally undergo assembly, retention or degradation in the endoplasmic reticulum [39]. Similar to Ig light chains [40], TFF1 obviously escapes this fate probably due to the four acidic residues flanking Cys^VII^ (Figure 1). Furthermore, Cys^VII^ is expected to be very nucleophilic because of its steric exposure by two proline residues in close proximity (Figure 1). Thus, Cys^VII^ would be ideally suited to serve as a scavenger for extracellular reactive oxygen/nitrogen species (ROS/RNS) [4,9,37,38]. In addition, TFF1 could also fulfill an intracellular function as a chaperone to ensure the correct folding and assembly of, for example, the gastric mucin MUC5AC [4,37,41]. Furthermore, minor amounts of TFF1 form disulfide-linked hetero-dimers with gastrokine 2 (GKN2) and IgG Fc binding protein (FCGBP) [9,33,37,42]. Finally, dimeric TFF1 has lectin activity toward both a core oligosaccharide of the *Helicobacter pylori* lipopolysaccharide as well as the carbohydrate moiety of the mucin MUC6 [37,43,44]. An αGlcNAc residue seems to be a common motif in these different structures, which is probably part of the recognition sequence of the lectin TFF1 [4].

TFF2 is a lectin recognizing specifically the O-linked GlcNAcα1→4Galβ1→R moiety of the mucin MUC6 [8,23,33,35,36,45]. Remarkably, the unusual α1,4GlcNAc-capped sugar moiety of MUC6 is evolutionarily conserved from frogs to humans [46,47]. The TFF2/MUC6 interaction has been shown to alter the viscoelastic properties of mucous gels [48] and physically stabilizes probably the inner adherent layer of the two-layered gastric mucus [4,8,49]. The stabilizing effect is even visible at the electron microscopic level [49]. Thus, in agreement with data from *Tff2*-deficient (*Tff2*^KO^) mice, TFF2 is expected to play a role in the innate immune defense of the gastric mucosa [4,20,49]. Of note, the α1, 4GlcNAc-capped sugar moiety of MUC6 also suppresses *H. pylori* growth [50].

In the intestine as well as saliva, TFF3 mainly occurs as a disulfide-linked hetero-dimer with FCGBP [34,51]. FCGBP is a repetitive, cysteine-rich glycoprotein (consisting of about 5400 amino acid residues) ubiquitously expressed in vertebrates and cephalochordates, where it is a characteristic secretory product of most mucin-producing cells (such as TFF3), and thus appears in the corresponding body fluids [47,52]. The molecular function of FCGBP has not been elucidated in detail. Generally, it is an early response gene after microbial infection and seems to play a role in the mucosal innate immune defense [20]; it likely regulates pathogen attachment and the clearing of microorganisms [53,54]. For example, FCGBP could bind IgG after its transcytosis via the neonanal Fc receptor (FcRn), and this complex could trap microbia, including viruses [55,56]. The hetero-dimerization of TFF3 and FCGBP could modulate the binding characteristics to microbia by a lectin activity of TFF3 [4,9,20]. A similar effect is expected for TFF1–FCGBP [9,37].

### 1.3. Pathological Expression of TFF Peptides: Links to Inflammation and Cancer

Soon after their discovery, ectopic expression of TFF peptides was detected in pathological conditions, particularly during chronic inflammation, such as gastro-esophageal reflux disease, Barrett esophagus, gastric and duodenal ulcers, diverticulitis, inflammatory bowel disease, pancreatitis, hepatholithiasis, cholecystitis, salpingitis, and inflammatory nasal polypi (for reviews, see [3,11,20,57]). These studies were mainly based upon histological results (immunofluorescence, immunohistochemistry, and in situ hybridization). In most of these cases, a glandular structure termed “ulcer-associated cell lineage” (UACL, also known as pyloric or pseudo-pyloric metaplasia) was described as the prominent site for TFF peptide synthesis [58]. For example, both TFF1 and TFF2 are ectopically expressed in Crohn’s disease [59]. Furthermore, TFF2 and TFF3 were detected after mucosal injury/ulceration; TFF2 was expressed early, whereas TFF3 was a late response gene clearly indicating a different regulation of TFF2 and TFF3 [60]. Strongly increased levels of all three TFF peptides were also observed in the bronchioalveolar lavage fluid from patients with chronic obstructive lung disease (COPD) [61].

Synthesis of TFF peptides is also dysregulated (up- or down-regulated) in different metaplasias [62,63], as well as malignancies (compilations: [11,12]). There are also multiple reports suggesting different roles for TFF peptides in tumor progression [1,64,65,66,67,68].

An acute inflammation is a defense mechanism of the immune system driven primarily by myeloid cells (e.g., macrophages). Macrophages are phagocytic cells of the innate immune system and they have remarkable plasticity. They have two states of polarized activation: classically activated (M1) and alternatively activated (M2) phenotypes. The latter is subdivided at least into three subtypes (M2a, M2b, and M2c) [69]. The activation and response of macrophages is controlled by subsets of differently polarized CD4^+^ T lymphocytes (Th1, Th2 and Th17 cells), each secreting signature cytokines and expressing a lineage-specifying transcription factor [70,71,72]. The type of immune response after injury or infection depends upon pathogen/danger-associated molecular patterns (PAMPs/DAMPs) and is characteristic for different pathogens (e.g., extracellular or intracellular bacteria, parasitic helminths, fungi, and viruses) [71]. These PAMPs and DAMPs, as well as ROS, are sensed by pattern-recognition receptors (PRRs), which are activators of the inflammasome and also direct triggers of (acute) inflammatory as well as regenerative processes (reparative inflammation) [73]. Furthermore, inflammation underlies many chronic and degenerative diseases. Of special note, most, but not all, chronic inflammatory diseases increase the risk of cancer [74,75]. An inflammatory environment is also a hallmark of cancer [76,77]. Thus, cytokines produced by activated immune cells are an important link between inflammation and cancer [78,79].

**Figure 2:**
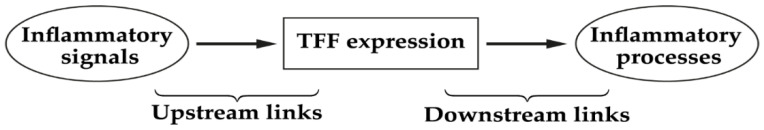
Schematic representation of the multiple links between TFF peptides and inflammation. TFF expression is regulated by inflammatory mediators (upstream); TFF peptides (or their loss) also influence inflammatory processes (downstream).

Numerous studies addressed the following question: which signals trigger the expression of TFF peptides during inflammation, and are there differences between the three TFF genes? Further questions asked were as follows: (i) is a loss of TFF peptides linked to inflammation, and (ii) is the expression of cytokines regulated by TFF peptides? The data concerning the multiple links of TFF peptides and inflammation are rather complex, partly seemingly controversial and often based upon single observations in a variety of very specialized systems. In order to get a glimpse on this complex interplay, the topic is discussed on two different levels (Figure 2): (i) complex regulation of TFF expression by inflammatory mediators (upstream links; Section 2); and (ii) role of TFF peptides in inflammatory processes (downstream links; Section 3). This scheme does not exclude possible feedback loops, e.g., those between inflammatory processes and the regulation of TFF expression (see Section 4.1).

In contrast to previous reviews describing the situation in healthy mucous epithelia (including the function of TFF peptides in the mucosal innate immune defense [4,20]), here, the role of TFF peptides during pathological, inflammatory conditions is discussed.

## 2. Regulation of TFF Expression by Inflammatory Mediators

Multiple reports indicate a complex regulation of TFF gene expression. Typical regulatory signals include estrogen, pro- and anti-inflammatory cytokines, transforming growth factor α (TGFα), fibroblast growth factors (FGFs), gastrin, TFF peptides (interregulation), prostaglandins, arachidonic acid, indomethacin, aspirin, omeprazole, butyrate, hydrogen peroxide, osmotic stress, hypoxia, X-ray irradiation, and pathogens (reviews: [11,65,66,80,81]). The three human TFF genes share some cis acting elements in their promoter regions [82]. Here, based on molecular data, the regulation of TFF gene expression during inflammatory conditions will be discussed for selected cases.

### 2.1. Down-Regulation of TFF1 during Gastric Inflammation and Ectopic TFF1 Expression in Chronic Inflammatory Diseases

An infection of the stomach with *H. pylori* is accompanied by gastritis, leading to dysregulated expression of TFF peptides. In the human antrum, on the protein level, mainly TFF1 is reduced in infected individuals [83]. In a mouse model of *H. pylori* infection, TFF1 expression is initially somewhat up-regulated transcriptionally and then also down-regulated about 14 days post-infection [84]. The down-regulation of TFF1 after *H. pylori* infection could be explained by a multi-step mechanism. First, *H. pylori*-infected cells (such as TFF1-secreting surface mucous cells) release interleukin (IL)-8 [85], which is a chemoattractant for neutrophils and macrophages. The latter then secrete IL-1β, which is the predominant pro-inflammatory cytokine produced in response to *H. pylori* infection; this shifts the immune response toward a Th1-axis (pro-inflammatory) [86]. From the in vitro data, one might conclude that IL-1β is responsible for the down-regulation of TFF1, as TFF1-3 expression is repressed by IL-1β (and IL-6) via nuclear factor κB (NF-κB) and CCAT/enhancer binding protein (C/EBP), respectively [87]. Of note, a similar down-regulation of TFF1 was observed also in other murine models of gastric inflammation [88]. Furthermore, TFF1 expression is also decreased in human gastric tissue along the multi-step cascade from inflammation and NF-κB activation to adenocarcinoma [89].

However, the situation concerning TFF1 expression during *H. pylori* infection is probably not that simple. For example, TFF1 expression (together with IL-8 expression) is strongly induced in vitro in the gastric adenocarcinoma cell line AGS after *H. pylori* infection [84,90]. Here, no immune cells are present, which would secrete IL-1β. One possible explanation would be that TFF1 expression is directly activated by *H. pylori* via ERK signaling [85,91]. Furthermore, there are indications that TFF1 suppresses *H. pylori*-induced gastric inflammation in vivo and in vitro [84,92].

In sharp contrast to the down-regulation of TFF1 in gastric inflammation, TFF1 expression is ectopically induced in different organs in chronic inflammatory diseases [59,93,94] as well as in different animal models of inflammation, such as encephalitis [95], asthma [62,96], pancreatitis [94], and in the murine spleen after *Toxoplasma gondii* infection [97,98]. The up-regulation of TFF1 was observed also in vitro in gastric epithelial cells by the pro-inflammatory Th1 cytokine tumor necrosis factor (TNF)-α via NF-κB [99]. Furthermore, the up-regulation of TFF1 expression during inflammation was described to occur via the transcription factor forkhead box (FOX) FOXA1 and FOXA2 (formerly: hepatocyte nuclear factors 3 α and β), which bind in human and rodent TFF1 promoters to motif IV, close to the TATA box [100]. These winged helix domain transcription factors play a role in acute-phase response and inflammatory processes [101]. Furthermore, the Th2 cytokine IL-13 also up-regulated TFF1 in bronchial epithelial cells in vitro and in an in vivo model; of note, FOXA2 was down-regulated and FOXA3 was up-regulated in this system [102]. In a murine asthma model, IL-13 seems to induce TFF1 expression in Clara cells (Clara cell metaplasia), which are able to trans-differentiate into goblet cells [62,96].

Indicative of the pleiotrophic nature of the cytokine IL-6, in vivo studies using mutated gp130 signal-transducing chains (*gp130*^757F^ and *gp130*^ΔSTAT^, respectively) for IL-6/IL-11 revealed that IL-6 can also potently positively regulate the expression of TFF peptides [81,91,103,104]. For example, in *gp130*^757F^ mutants, SHP2-Ras-ERK signaling is blocked, the Tff1 level is decreased, and antral adenomas and carcinomas are developed [104]. This is remarkably similar to the phenotype of *Tff1*^KO^ (development of antral adenomas and partly carcinomas [105]). Thus, TFF1 expression seems to require IL-6-triggered SHP2-Ras-ERK signaling [81,91,103,104].

Minute amounts of TFF1 are also synthesized in the brain, for example, in astrocytes [12]. In the latter, TFF1 expression can be induced in vitro by IL-6, IL-7, and TNF-α [106]. TNF-α has been shown to up-regulate TFF1 expression via NF-κB [99]. Furthermore, TFF1 (but not TFF2 or TFF3) is up-regulated in two murine encephalitis models, probably in neurons (e.g., in internal granular layer of the cerebellum) [95]. Both models are accompanied by a strongly increased expression of TNF-α [95].

TFF1 (but not TFF2 or TFF3) is also up-regulated in the immune system, e.g., in the murine spleen after *T. gondii* infection (two models) [97,98]. Here, TNF-α is also up-regulated [97], which could be responsible for the induced TFF1 transcription via NF-κB [99]. Furthermore, the specific up-regulation of TFF1, but not of TFF2 and TFF3, could also be induced by the binding of FOXA1 and FOXA2 to motif IV in the TFF1 promoter [100].

### 2.2. Regulation of TFF2 during Inflammation

In contrast to TFF1, TFF2 was only transiently reduced in the human stomach after *H. pylori* infection [83]. TFF2 is rather up-regulated in inflammatory conditions as shown for various diseases [107], as well as in murine models of gastric inflammation [88] and allergic airway disease [108]. For example, gastrin-deficient mice exhibit chronic inflammation in the hypochlorhydric stomach and the Th1 cytokine interferon-gamma (IFN-γ) is the most abundant pro-inflammatory cytokine [109]. Using the gastric cell line NCI-N87, TFF2 expression was induced by IFN-γ [109]. In MKN45 gastric cells, the nuclear peroxisome-proliferator-activated receptor γ (PPARγ) regulates TFF2 expression via a non-canonical response element (PPRE) [110]; other than typical PPARγ ligands, such as troglitazone, non-steroidal anti-inflammatory drugs (NSAIDs), such as indomethacin, can induce TFF2 expression by activating PPARγ [110].

Furthermore, TFF2 was strongly induced in the lung in murine asthma models by the Th2 cytokines, IL-4 and IL-13 [111]. TFF2 induction can occur in both a STAT6-dependent manner (by IL-4, IL-13, and ovalbumin) and a STAT6-independent mechanism (by chronic expression of IL-4 or by the allergen *Aspergillus fumigatus*) [111]. The Th2 cytokine-mediated induction of TFF2 expression probably occurs via an indirect mechanism, as the TFF2 promoter is not known to contain a STAT-binding site but is rather regulated via GATA6 [111]. TFF2 was also induced in vivo in the murine lung as well as in vitro in human bronchial epithelial cell cultures by IL-13 [102]. Thus, TFF2 seems to be inducible during inflammation in different ways, i.e., by the Th2 cytokines IL-4 and IL-13 as well as by allergens.

Minute amounts of TFF2 are also expressed in the immune system, such as the thymus, bone marrow, spleen (memory T cells), lymph nodes, and peritoneal macrophages [14,15,16,17,18]. In the rat spleen, there is a biphasic regulation of TFF2 (up-regulation starting at 96 h) following lipopolysaccharide (LPS) administration; the latter induces an inflammatory reaction [14].

### 2.3. Regulation of TFF3 during Inflammation

The Th2 cytokines IL-4 and IL-13 up-regulate TFF3 expression in vitro via the transcription factor STAT6 [112]. Of special note, the heterodimer partner of TFF3, i.e., FCGBP, is also up-regulated by IL-13 [102,113]. This points to a co-ordinate expression of these disulfide-linked partner proteins during Th2 inflammation. Furthermore, in rodent models, TFF3 expression is increased in the colon after infection with pathogens, such as *Nippostrongylus brasiliensis* [114], *Bifidobacterium dentium* [115], and co-infection with *Giardia muris* and *Citrobacter rodentium* [116]. The latter is dependent on the NLRP3 inflammasome [116]. In contrast, infection with *Citrobacter rodentium* alone reduces TFF3 expression and *Rag*^KO^ (T and B cell-deficient) mice did not exhibit this reduction [116,117]. Thus, it is mainly the host immune system that modulates the function of the goblet cells [117].

In contrast, the Th1 cytokine TNF-α inhibits TFF3 expression via NF-κB [118]. TFF3 repression occurs via promoter binding sites for NF-κB and C/EBPβ [118,119]. Furthermore, IL-1β and IL-6 and a combination of both can also down-regulate TFF3 expression in certain cell lines [87].

On the other hand, mice with a mutated gp130 signal-transducing chain (*gp130*^ΔSTAT^) of the IL-6/IL-11 receptor had a reduced Tff3 level and impaired intestinal wound healing [91]. This phenoptype is remarkably similar to that of *Tff3*^KO^ mice [120]. Thus, in this in vivo model, TFF3 expression seems to depend on IL-6-triggered STAT1/3 signaling [81,91,103].

TFF3 is also linked to the intestinal innate immune response as its expression is induced after activation of Toll-like receptor 2 (TLR2) by commensal bacteria [121]. This is probably a secondary effect, as goblet cells probably do not express TLR2. Of special note, a severe form of ulcerative colitis (pancolitis) is associated with the heterozygous TLR2-R753Q polymorphism [122], which failed to induce TFF3 synthesis, at least in vitro [121].

Minor amounts of TFF3 are expressed in lymphatic organs such as the thymus and bone marrow as well as the spleen (memory T cells) and lymph nodes [14,15,123]. In the murine thymus, TFF3 expression is up-regulated by the autoimmune regulator (Aire) [123]. In the rat spleen, there is a biphasic regulation of TFF3 (up-regulation starting at 14 h) after exposure to LPS [14].

In the rodent and human brain, minute amounts of TFF3 are expressed mainly in neurons and also in the choroid plexus, but not in astrocytes or resting microglial cells [124,125,126,127]. Of note, in rodent primary cultures, TFF3 expression was detected in neurons as well as in activated microglial cells, but not in astrocytes [126]. The expression in activated microglial cells points to a neural immune function of TFF3, as these cells are the resident myeloid cells of the CNS, forming its innate immune defense [128].

## 3. Role of TFF Peptides for Inflammatory Processes

Generally, the role of TFF peptides in influencing inflammatory processes can be investigated by loss-of-function models (e.g., various *Tff*-deficient mice) and by gain-of-function studies (e.g., direct application of TFF peptides). Of special note, there is a remarkably limited number of convincing reports describing significant effects of TFF peptides (e.g., in vitro) in gain-of-function studies; there are specific and sensitive readouts missing, which would allow direct functional measurements. This might be a further indication that the major functions of TFF peptides probably do not rely on simple ligation to high-affinity transmembrane receptors and triggering signaling cascades.

### 3.1. Loss of TFF1 Is Linked to Antral Inflammation and Cancer

*Tff1*^KO^ mice, in contrast to *Tff2*^KO^ und *Tff3*^KO^ mice, have a severe phenotype, i.e., they all develop adenomas in the gastric antral and pyloric mucosa and about 30% progress to carcinomas [105,129]. As early as 3 days postnatally, pits and glands in the antropyloric region are elongated due to severe hyperplasia and there is an expansion of proliferating epithelial progenitor cells, the latter being almost entirely devoid of mucus [129,130]. Interestingly, *gp130*^757F^ mutants with blocked SHP2-Ras-ERK signaling of the IL-6/IL-11 receptor show strongly reduced *Tff1* levels and a similar phenotype [104]. In *Tff1*^KO^ mice, *Tff2* expression is also drastically reduced, particularly in the gastric corpus, but not so much in the pancreas [9,129,131]. From results with *gp130*^757F^ mutants and *gp130*^757F^/*Tff2*^KO^ mice [132], one might conclude that the reduced Tff2 level in *Tff1*^KO^ mice probably exacerbates antral tumorigenesis. The loss of TFF1 is associated with activation of NF-κB-mediated chronic antral inflammation and multi-step carcinogenesis [89]. This is accompanied by an increased level of T lymphocytes and dramatic induction of IL-17 expression with age [133]. Of special note, the selective Cox-2 inhibitor celecoxib significantly reduced dysplastic lesions, clearly demonstrating the consecutive link of chronic inflammation and carcinogenesis [89,105,134]. Thus, *Tff1* is a gastric tumor suppressor in mice [105]. In addition, the observation that TFF1 triggers a delay of the cell cycle [135] is typical of tumor suppressors. Furthermore, *Tff1*^KO^ mice show significantly higher tumor incidence after chemically-induced tumorigenesis [136].

Interestingly, at 5 months, the villi of the small intestinal mucosa were enlarged (hyperplasia) in *Tff1*^KO^ mice by a thickened lamina propria, which contained inflammatory cells [129]. A role of TFF1 outside the stomach is in line with lineage tracing studies using *Tff1-Cre* mice, which detected labeling also in the intestine [137]. However, *Tff1*^KO^ mice did not show an increased susceptibility to dextran sulfate sodium (DSS)-induced colitis [17].

Lineage tracing studies using transgenic *Tff1*-*CreERT2* and *Tff1-Cre* mice showed that *Tff1* is also expressed in long-lived stem and progenitor cells of the gastric antrum, which finally re-populate the entire antral units [137,138]. In contrast, the fundic units were only partially traced by these cells [137,138]. This is surprising and remarkable. Fundic and antral units undergo continuous self-renewal from stem and progenitor cells, but the progenitor cells differ characteristically in these units (for review, see [139]). The clonal expansion in single glands is more rapidly in the antrum when compared with the corpus [140]. Fundic units mainly contain *Troy*^+^ progenitor cells at their base [141], whereas at the base of antral units, mainly *Lgr5*^+^ progenitor cells are found, which probably originate from *Cckbr^+^* progenitor cells at the +4 position [142,143]. Thus, the study with the *Tff1*-*CreERT2* and *Tff1-Cre* mice would explain why inflammation and carcinogenesis in *Tff1*^KO^ mice are restricted to the antrum, as *Tff1* is expressed possibly already in *Lgr5*^+^ (and maybe also in *Cckbr^+^*) progenitor cells, but not in fundic *Troy*^+^ progenitor cells [138]. This is also in line with the significant up-regulation of *Cckbr* and the transcription factor *Mist,* specifically in the gastric antrum of *Tff1*^KO^ mice [9].

Finally, the question arises on the precise molecular function of TFF1 and how a loss of TFF1 triggers gastric inflammation and carcinogenesis. Currently, at least four hypothetical models (or a combination of these) are plausible.

First, TFF1 could be an intracellular chaperone, as, in *Tff1*^KO^ mice, the unfolded protein response (UPR) is activated [41,105]. This is in agreement with the discovery of a disulfide-linked TFF1 heterodimer with a yet unknown partner protein X (TFF1-X; M_r_ of 60k) in the human stomach; X might be a disulfide isomerase of the endoplasmic reticulum (ER) related to ERp57 [4,37]. ERp57 is not only involved in the correct folding of glycoproteins and assembly of the major histocompatibility complex (MHC class I), but also regulates gene expression via interaction with STAT3 [144]. Of note, the expression of the ER disulfide isomerase *Pdia3* (i.e., the murine homologue of human ERp57) is significantly up-regulated in the gastric fundus and antrum of *Tff1*^KO^ mice [9]. This model is also in line with the observation that lectins play an important role in quality control and glycoprotein sorting in the secretory pathway [145].

Second, TFF1 was postulated to act as a scavenger for extracellular ROS/RNS due to its exposed and probably highly nucleophilic Cys^VII^ residue [4,9,37,38]. Such protection is of particular importance for the gastric mucosa, as it is the target, as well as a potent generator, of ROS/RNS [4]. In particular, stem cells are highly sensitive to damage by ROS. An ultimate test of this hypothesis would be to check if a synthetic peptide mimicking the C-terminal Cys^VII^ of TFF1 cures *Tff1*^KO^ mice from developing adenomas and carcinomas.

Third, TFF1 could serve as an extracellular lectin, recognizing a yet not identified glycoprotein with a terminal GlcNAcα1→R moiety or a similar structure. This unusual sugar moiety is characteristic of the mucin MUC6 from frog to human and is essential for binding the lectin TFF2 [8,36]. The addition of the terminal GlcNAcα residue is catalyzed by the enzyme α1,4-*N*-acetylglucosaminyltransferase (α4GnT) [146]. Remarkably, *A4gnt*^KO^ mice have a very similar phenotype to *Tff1*^KO^ mice [146]. Recently, dimeric TFF1 has also been shown to bind to MUC6 as a lectin; the terminal GlcNAcα moiety or a similar structure is likely involved in this binding [37,44]. Thus, one might speculate that the ligation of TFF1, or even a modified TFF1 (e.g., sulfenylated TFF1), to MUC6 or a yet not identified transmembrane glycoprotein could serve as a signal for the correct self-renewal of antral units. Generally, TFF1 could act as an activating, as well as an inhibitory, ligand (see also Section 4.1). The latter possibility is increasingly interesting, as TFF1 has been shown to block the interaction of the IL-6 receptor IL6Rα-gp80 and gp130 (signal-transducing chain) [147], and maybe also the interaction of TNF-α and its receptor [89]. However, the known lectin interaction of MUC6 and TFF2 does not seem to play a role here, as *Tff2*^KO^ mice have a completely different phenotype to *A4gnt*^KO^ mice.

Fourth, a 37k-entity of Gkn2, probably a Gkn2 homodimer, was recently detected in *Tff1*^KO^ mice, only (particularly in the antrum) [9]; the usual Tff1-Gkn2 heterodimer cannot be synthesized any more in these mice because of a lack of Tff1. Such a secretory Gkn2 homodimer may impact the inflammatory processes in the antrum or influence early differentiation. In human, the major amounts of GKN2 are hardly soluble and are probably part of the inner gastric mucus layer [37].

### 3.2. TFF2: Component of the Gastric Mucus Barrier (Lectin Binding to MUC6), Inhibition of Myeloid Cells (Anti-Inflammatory Factor), and Increased Synthesis of the Alarmin IL-33 (Promotion of Th2 Immunity)

*Tff2*^KO^ mice did not show obvious gastrointestinal abnormalities [148]. However, in *Tff2*^KO^ mice, the degree of gastric ulceration after administration of the COX1/2 inhibitor indomethacin was significantly increased [148] and the recovery of the gastric surface from laser-induced photodamage was delayed [149]. *Tff2*^KO^ mice also exhibited accelerated progression of gastritis to dysplasia in the gastric antrum after infection with *H. pylori* [150] and these animals show an increased susceptibility to *H. felis*-induced gastritis, with enhanced gastric inflammation [16]. All these effects are in agreement with a hypothetical function of TFF2 in the gastric mucosal innate immune defense by physically stabilizing the inner mucus barrier layer due to its lectin interaction with MUC6 [4,8,20].

TFF2 expression is not limited to the gastrointestinal tract but is also present in macrophages and lymphocytes. Remarkably, peritoneal macrophages from *Tff2*^KO^ mice were hyperresponsive to IL-1β stimulation concerning the secretion of IL-6 [16]. Thus, TFF2 functions as an anti-inflammatory peptide in immune cells, negatively regulating the expression of IL-1β-induced genes. This in vitro result might be in agreement with an in vivo study, where colonic IL-6 production was dramatically reduced in a murine DSS colitis model after topical pretreatment (intracolonic route) with TFF2 [151]. A similar protective effect against DSS-induced colitis was also obtained with a TFF2-secreting *Lactococcus lactis* strain, which had a therapeutic effect even in chronic colitis in *Il10*^KO^ mice [152]. In contrast, *Tff2*^KO^ mice exhibited a more severe response to and a delayed recovery from DSS-induced colitis [16,17]. A conclusive explanation is not possible currently as, in *Tff2*^KO^ mice, colonic *Tff3* expression is also strongly reduced, which could be the cause of this phenotype [17]. Surprisingly, the protective effect of TFF2 from DSS-induced colitis seemed to originate from colonic epithelial cells and not from colonic leucocytes, as TFF2 is not synthesized in the latter [17].

In another animal model of intestinal inflammation, *Tff2*^KO^ mice were orally infected with *T. gondii* [153]. In wild type mice, this leads to lethal ileitis. Surprisingly, *Tff2*^KO^ mice showed an increased baseline level of IL-12/23p40 when compared with the wild type, but they did not develop the typical intestinal immunopathology [153]. Generally, TFF2 antagonized the IL-12 release from macrophages and dendritic cells [153]. This inhibitory effect was due to cell-intrinsic TFF2 expression and could be also induced by exogenous TFF2 [153]. IL-12 is a known driver of Th1 inflammation, leading to a preferential expansion of IFN-γ-producing lymphocytes. Of note, in *Tff2*^KO^ mice, the baseline production of IFN-γ was not different, but the expansion of IFN-γ-producing Th1 cells was greatly induced after *T. gondii* infection [153]. Taken together, in this animal model, TFF2 is an anti-inflammatory peptide, down-regulating the expression of IL-12 in macrophages and dendritic cells, leading to a suppression of the Th1 immune response after *T. gondii* infection. Currently, the precise molecular mechanism of how TFF2 inhibits TLR-driven IL-12 expression is not known. A receptor blocking mechanism may be involved, as discussed in Section 4.1.

In contrast to infection with *T. gondii* [153], oral infection with *Yersinia enterocolitica* resulted in a lethal outcome in *Tff2*^KO^ mice, but not in wild type mice [154]. In *Tff2*^KO^ mice, the reduced amount of macrophages allowed *Y. enterocolitica* to cross the epithelial barrier of the ileum [154]. Currently, a proper explanation of these results it is not possible as there are no more molecular data available. The reduced *Tff3* synthesis in *Tff2*^KO^ mice [17] may also contribute to this result.

In another set-up, nine day-old (P9) *Tff2*^KO^ rats were orally infected with *E. coli*, which led to bacteremia, in contrast to the wild type [155]. At this time point, intestinal *Tff2* expression reaches a peak and drops sharply thereafter [156]. Thus, the increased susceptibility of *Tff2*^KO^ rats is in agreement with a function of TFF2 for the barrier integrity of the neonatal rat intestine.

In a further study, TFF2 from splenic memory T cells suppressed the expansion of splenic myeloid-derived suppressor cells (MDSC) via CXCR4 [18]. The number of MDSCs is increased in tumors where they create an inflammatory environment. *Tff2*^KO^ mice had an increased number of MDSCs and exhibited a greater number of tumors in an azoxymethane/DSS model of inflammatory colorectal carcinogenesis [18].

The inhibitory effect of TFF2 on macrophages was also demonstrated by a myeloid-specific deletion of *Tff2* (*Cd11c*^Cre^*Tff2*^flox^ mice) [157]. After infection with the hookworm *Nippostrongylus brasiliensis*, the lung pathology was exacerbated in these mice and the proliferative expansion of epithelial alveolar type 2 cells was reduced [157]. The latter was due to the diminished expression of *Wnt4* and *Wnt16*. Thus, myeloid-derived TFF2 also drives macrophages to accelerate epithelial regeneration after lung injury [157].

After the infection of mice with *N. brasiliensis*, TFF2 expression increased first in the lung (early stage) and then in the intestine (late stage); this is a prerequisite for the induction of IL-33 production, a Th2-promoting cytokine, in lung epithelial cells, alveolar macrophages, and inflammatory dendritic cells [158]. Thus, in parasitized *Tff2*^KO^ mice, the IL-33 levels are only slightly increased [158]. Of special note, the TFF2-triggered induction of IL-33 synthesis in bone marrow-derived macrophages required CXCR4 [158], which is a putative TFF2 receptor [26,27].

The TFF2-IL-33 axis has also been described in the stomach, where IL-33 is synthesized in a subpopulation of surface mucous cells, probably in precursors of surface mucous cells [159]. In the CNS and other epithelial tissues, IL-33 is expected to act as an alarmin by responding rapidly after insult [159]. In *Tff2*^KO^ mice, IL-33 expression is significantly reduced at least in the gastric fundus [159]. Furthermore, *H. pylori* infection also changed IL-33 expression biphasically—an acute phase with increased IL-33 followed by suppression in the chronic phase [159]. Chronic IL-33 application caused an infiltration of macrophages, neutrophils, and dendritic cells into the stomach, leading to a Th2 immune response as well as activation of the already present group 2 innate lymphoid cells (ILC2), particularly in the antrum [159]. Taken together, one could postulate that exocrine epithelial TFF2 might induce IL-33 expression in gastric surface mucus cells after injury and disruption of the gastric mucosal barrier, allowing ligation of a putative basolateral TFF2 receptor, such as CXCR4. A similar activation of a basolateral receptor after injury has been described for heregulin-α and its receptor in epithelial cells of the lung [160].

**Figure 3:**
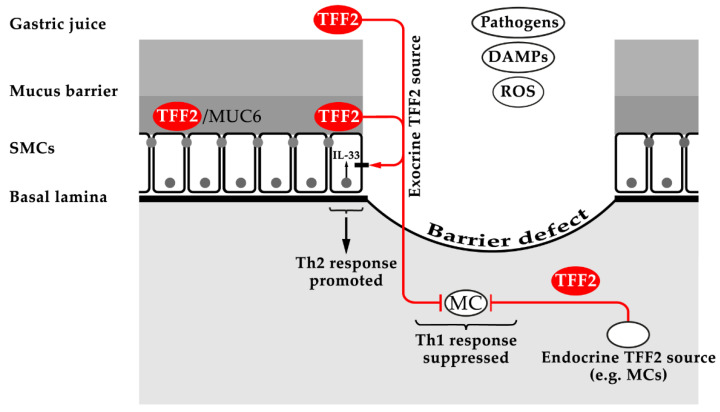
Schematic representation of the putative functions of TFF2 in the normal gastric mucosa as well as after injury/infection. The TFF2/MUC6 complex probably stabilizes the inner gastric mucus barrier layer at the apical side of surface mucous cells (SMCs); the mucus as well as the luminal content of the stomach are separated by tight junctions from the basolateral side of SMCs. After a barrier defect, exocrine TFF2 might stimulate a putative TFF2 receptor (such as CXCR4) at the basolateral surface, leading to an increased synthesis of the nuclear alarmin IL-33 and promotion of a Th2 response (after IL-33 release probably by a non-classical secretory mechanism via exosomes [161]). Furthermore, exocrine TFF2 as well as TFF2 from endocrine sources probably have an inhibitory effect on myeloid cells (MC; see also Section 4.1), repressing a Th1 response.

Taken together, TFF2 has a function in the normal stomach as a constituent of the gastric mucus barrier (physical stabilization of the inner, insoluble layer by strong lectin interaction with MUC6), which is a first line defense against microbial infections (innate immunity; Figure 3) [4,8,20]. In contrast, after injury or infection, TFF2 has diverse roles in the immune system and for inflammation. This explains why *Tff2*^KO^ mice have a compromised immune system [15]. On the one hand, TFF2 is a brake for myeloid cells (e.g., inhibition of IL-6 and IL-12 release) so that, in particular, Th1 inflammation after a mucosal challenge (infection) is not overshooting (anti-inflammatory effect; Figure 3) [16,18,107,153]. On the other hand, TFF2 is a positive regulator of the alarmin IL-33 in the CNS and mucous epithelia, which is an activator of a Th2 immune response after injury (Figure 3) [158,159].

Currently, it is not clear how TFF2 triggers the immune modulatory effects in the different cell types. There are multiple indications that one putative receptor is CXCR4 [18,26,27,158,162]. However, currently there are no data defining the interaction of TFF2 and CXCR4 (lectin or a protein–protein interaction). As TFF2 is a lectin, binding strongly to the O-linked GlcNAcα1→4Galβ1→R moiety of the mucin MUC6 [8,23,36,45], an interaction of TFF2 with the carbohydrate moiety of CXCR4 would be not surprising. Such a lectin interaction could also have the advantage of being specific for a cell type, depending on the glycosylation status of the cell [20]. Furthermore, signaling by TFF2 could also be more complex, e.g., by binding to glycosaminoglycans, as shown for a number of cytokines [163].

### 3.3. Loss of Tff3 Is Linked to Increased DSS-Induced Colonic Inflammation

*Tff3*^KO^ mice develop normally and are grossly indistinguishable from their wild type littermates [120]. However, the migration of colonic crypt cells due to self-renewal of the epithelium from precursor cells was strongly delayed [120]. In the DSS-induced colitis model (2.5% DSS), *Tff3*^KO^ mice reacted much more sensitively when compared with the wild type animals [120]. Of special note, also a number of mouse strains with reduced TFF3 levels showed a similar phenotype in the DSS colitis model to the *Tff3*^KO^ animals: *Agr2*^KO^ [164], *Tff2*^KO^ [17], and *gp130*^ΔSTAT^ [91].

The murine colonic mucus consists of two layers: a firmly adherent inner layer, and a loose outer layer. Normally, the inner layer is devoid of bacteria [165,166]. After DSS treatment, the thickness of the inner mucus layer of the colon decreased and became permeable so that bacteria were able to penetrate and reach the epithelial cells even after 4 h [167]. This occurred before infiltration of the immune cells was observed. In wild type mice, the TFF3 expression was increased after DSS treatment in an early phase [168,169]. Thus, the increased sensitivity of *Tff3*^KO^ mice in the DSS colitis model is probably an indication that in these animals, more bacteria reach the epithelium due to an intestinal mucosal barrier defect. Most of the intestinal TFF3 forms a hetero-dimer with FCGBP, which is mucus-associated [34] and is expected to play a role in the mucosal innate immune defense by, for example, regulating pathogen attachment and the clearing of microorganisms [20]. It would be interesting to test if *Tff3*^KO^ mice show also mucosal barrier defects in the oral cavity or the urogenitary tracts, as TFF3 (and FCGBP) is also synthesized in these epithelia. Another interesting goal would also be the generation of *Fcgbp*^KO^ mice and to determine their phenotype in the DSS colitis model. Furthermore, the binding of TFF3 to DMBT1^gp340^, a pattern recognition receptor with a function in mucosal innate immunity, could play a protective role here [20,25,170].

Of note, the expression of pro-inflammatory cytokines in cultured microglial cells was reduced by TFF3 [171]. This points to an anti-inflammatory function of TFF3 by the shifting of microglial cells from a M1 to a M2 phenotype, at least in vitro [171].

An immunomodulatory role of TFF3 is also in line with the observation that in the murine spleen after *T. gondii* infection, the expression of the inflammasome constituent *Nlrp12* was significantly reduced in *Tff3*^KO^ mice when compared with wild type mice [98].

## 4. Conclusion and Medical Perspectives

Taken together, from loss-of-function studies, it is clear that *Tff*-deficient mice have completely different phenotypes, but all are related to inflammatory processes, either directly or after various mucosal challenges. The following picture concerning the multiple and different functions of TFF peptides has emerged (Table 1):

Under physiological, healthy conditions, TFF peptides fulfill their protective functions as exocrine products mainly in the gastric mucosa (TFF1, TFF2), or in a variety of mucous epithelia (TFF3). Here, they play a role as a gastric tumor suppressor (TFF1) or they are involved in the mucosal innate immune defense as integral parts of the mucus barrier (TFF2/MUC6 lectin complex, TFF3-FCGBP heterodimer) [4,20]. As a hallmark, all three TFF peptides have lectin activities, best characterized for TFF2 [4,22]. Thus, TFF peptides as soluble lectins are comparable with multifunctional galectins and C-type lectins, which also interact with mucins [172,173]. Currently, it cannot be excluded that TFF peptides also recognize microbial glycans as certain soluble lectins do [174]. Generally, TFF peptides act at the delicate interface of epithelia, mucus/mucins, and microbia. Here, a number of medical applications are within the limits of expectation; a porcine gastric mucin preparation is already used as artificial saliva, which contains TFF2 [36]. Similar topical formulations could be used to treat patients with gastric or duodenal ulcers [20]. Equally promising are luminal applications of TFF3-FCGBP or TFF3/DMBT1gp340 for the treatment of various infections of mucous epithelia (development of anti-bacterial and anti-viral formulations) [20].

Under pathological conditions, e.g., after mucosal injury or infection, TFF2, in particular, is secreted in an endocrine fashion by myeloid cells (e.g., macrophages) and lymphocytes (e.g., memory T cells). Here, at least TFF2 is a modulator of immune reactions triggering inflammatory processes. On the one hand, TFF2 induces the synthesis and release of the nuclear alarmin IL-33, at least in mucous epithelia, which leads to a Th2 immune response. There are multiple indications that certain TFF2 effects are mediated by activating ligation to CXCR4 and/or a plethora of other glycosylated transmembrane proteins. Unfortunately, the details are not known currently. As TFF2 is a lectin recognizing, at least, the O-linked GlcNAcα1→4Galβ1→R moiety of the mucin MUC6, a lectin-triggered activation of a glycosylated transmembrane protein seems reasonable [23]. On the other hand, TFF2 inhibits myeloid cells.

### 4.1. Lectin-Triggered Receptor Blocking by TFF Peptides: An Hypothesis

**Figure 4:**
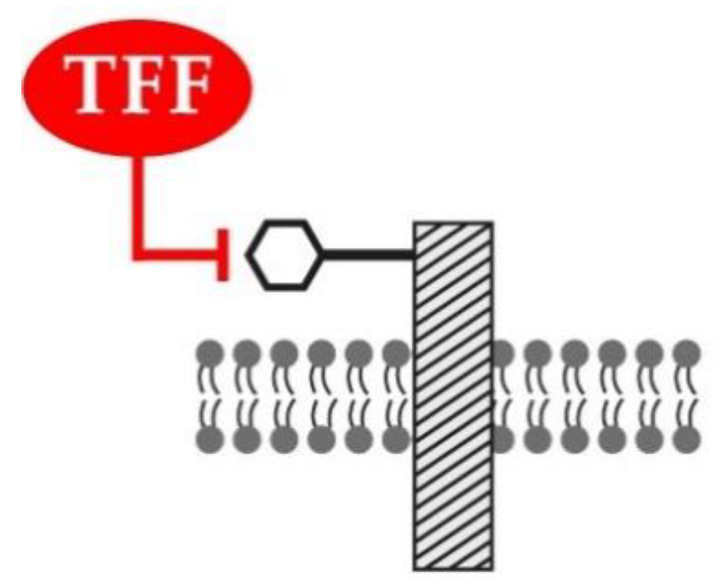
Lectin-triggered receptor blocking hypothesis. TFF peptides are proposed to bind as lectins to the carbohydrate moiety (indicated by a hexagon) of a glycosylated transmembrane receptor (hatched rectangle), thus negatively interfering with the binding of the natural ligand(s).

In contrast to a proposed lectin-triggered activation of receptors [23], TFF peptides can, in particular, block the ligation of natural ligands and their cognate membrane receptors. This has been demonstrated for TFF1, which blocks the interaction of IL6Rα-gp80 (IL-6 binding chain) and gp130 (signal-transducing chain) in vitro by interaction with IL6Rα-gp80 [147]. As a consequence, several STAT3 target genes are overexpressed in *Tff1*^KO^ mice [147]. The precise nature of TFF1 binding to IL6Rα-gp80 has not been elucidated thus far, but it is tempting to speculate that TFF1 acts as a lectin binding to the carbohydrate moiety of IL6Rα-gp80, which blocks receptor activation (lectin-triggered receptor blocking hypothesis; Figure 4).

Accordingly, TFF1 is a natural antagonist of the IL-6 receptor system and comparable with the action of tocilizumab, a humanized anti-IL-6 receptor antibody, which is used in the clinics for treating rheumatoid arthritis, cytokine release syndrome and even COVID-19 [175]. A similar situation might occur in the TNF-α receptor system, where TFF1 suppressed TNF-α-mediated NF-κB activation through TNFR1 [89] and inhibited the expression of the tissue inhibitor matrix metalloproteinase-1 (TIMP1) [176]. Furthermore, the hyperresponsiveness of peritoneal macrophages from *Tff2*^KO^ mice to IL-1β stimulation [16] might be due to the lectin binding of TFF2 to the IL-1 receptor. Such an inhibition of the IL-1 receptor system by TFF2 is reminiscent of the IL-1 receptor antagonist anakinra, which is clinically used for treating rheumatoid arthritis.

### 4.2. New Medical Perspectives

In the future, TFF peptides might be used to specifically block a series of glycosylated receptors playing mayor roles in inflammatory processes. As glycosylation patterns are relatively cell-specific, the use of TFF peptides could be selective for specific cells. Furthermore, TFF peptides recognize different carbohydrate moieties. This combination might allow interesting future clinical applications for TFF peptides and might open new therapeutic strategies, e.g., as anti-inflammatory agents. Thus, it is now a promising goal to test systematically receptors for their binding of TFF peptides, e.g., in vitro. When using different cell lines, the knowledge of the specific glycosylation pattern is of particular interest. The use of different cell lines might explain contrary past results. As a prerequisite for such studies, the carbohydrate specificities of TFF peptides have to be elucidated in detail. Thus far, it is clear that the lectin characteristics of the three TFF peptides are different, but partly related. GlcNAc seems to be a common moiety recognized at least by TFF1 and TFF2 [4]. Furthermore, a series of mutant TFF peptides with precisely altered carbohydrate specificities could be created, which would even expand their medical potential [4,20].

As lectins with multiple connections to the immune system, TFF peptides have to be considered as promising new players in the field of glycoimmunology, such as galectins, siglecs, and C-type lectins [177,178]. In particular, galectins are able to form multivalent complexes with cell surface glycoconjugates, and such 2D and 3D cross-linked lattices could influence signal transduction [179,180]. As a prerequisite for such studies, it has to be cleared which of the polarized macrophage phenotypes and which lymphocytes synthesize which TFF peptide. In addition, it is also important to understand the rather complex regulation of TFF gene expression in these cells. For example, PPARγ is involved in the regulation of macrophage polarization [181] as well as TFF2 expression [80,110]. Furthermore, there are auto-induction mechanisms known for TFF genes [80,182] as well as epigenetic regulation via promoter methylation [11,66,80,132,183,184,185]. Such mechanisms would be well suited for generating positive and negative feedback loops. This information is necessary not only to fully understand the numerous results particularly obtained with the *Tff2*^KO^ mice (see Section 3.2), but also for a rationale clinical application of TFF peptides.

First clinical studies already started with TFF1 and TFF3 only (probably for patent reasons) to reduce oral mucositis, which is a side effect of radio- and chemotherapy [186]. For the future, more sophisticated strategies can be expected, which could allow numerous novel medical applications for TFF peptides; possible fields would be inflammation-induced fibrosis, rheumatoid arthritis, or neurodegeneration. For example, TFF2 regulates airway remodeling [187], reversed airway fibrosis [108], and is upregulated in the synovial fluid of rheumatoid arthritis samples [188], whereas TFF3 is associated with neurodegeneration [189]. However, there are many open questions currently and there is a strong necessity for further research before application in clinics can be considered, e.g., for the treatment of various immune mediated inflammatory disorders.

## Figures and Tables

**Table 1:** TFF peptides and their downstream links to inflammation

Loss of TFF	Impaired Functions	Inflammatory Phenotypes
TFF1	Dysregulated self-renewal of gastric antral units	Antral inflammation and cancer
TFF2	Gastric barrier defect	Enhanced gastric inflammation after *H. pylori* infection Changed inflammatory responses after infections
	Dysregulated immune reactions	
TFF3	Intestinal barrier defect	Increased inflammation after DSS challenge
	Dysregulated immune reactions?	Changed inflammatory responses?

## Abbreviations

CNS	Central nervous system
DAMPs	Danger-associated molecular patterns
DSS	Dextran sulfate sodium
ER	Endoplasmic reticulum
FCGBP	IgG Fc binding protein
FOX	Forkhead box
GKN	Gastrokine
IFN	Interferon
IL	Interleukin
NF-κB	Nuclear factor kappa B
PAMPs	Pathogen-associated molecular patterns
ROS	Reactive oxygen species
SDS	Sodium dodecyl sulfate
SMC	Surface mucous cell
TFF	Trefoil factor family
TGF	Transforming growth factor
TLR	Toll-like receptor
TNF	Tumor necrosis factor

## References

[1] Ribieras S., Tomasetto C., Rio M.C. (1998). The pS2/TFF1 trefoil factor, from basic research to clinical applications. Biochim. Biophys. Acta.

[2] Thim L., May F.E.B. (2005). Structure of mammalian trefoil factors and functional insights. Cell. Mol. Life Sci..

[3] Kjellev S. (2009). The trefoil factor family—small peptides with multiple functionalities. Cell. Mol. Life Sci..

[4] Hoffmann W. (2020). Trefoil factor family (TFF) peptides and their diverse molecular functions in mucus barrier protection and more: Changing the paradigm. Int. J. Mol. Sci..

[5] Wright N.A., Hoffmann W., Otto W.R., Rio M.C., Thim L. (1997). Rolling in the clover: Trefoil factor family (TFF)-domain peptides, cell migration and cancer. FEBS Lett..

[6] Thim L. (1989). A new family of growth factor-like peptides. ‘Trefoil’ disulphide loop structures as a common feature in breast cancer associated peptide (pS2), pancreatic spasmolytic polypeptide (PSP), and frog skin peptides (spasmolysins). FEBS Lett..

[7] Hoffmann W., Hauser F. (1993). The P-domain or trefoil motif: A role in renewal and pathology of mucous epithelia?. Trends Biochem. Sci..

[8] Heuer F., Stürmer R., Heuer J., Kalinski T., Lemke A., Meyer F., Hoffmann W. (2019). Different forms of TFF2, a lectin of the human gastric mucus barrier: In vitro binding studies. Int. J. Mol. Sci..

[9] Znalesniak E.B., Salm F., Hoffmann W. (2020). Molecular alterations in the stomach of Tff1-deficient mice: Early steps in antral carcinogenesis. Int. J. Mol. Sci..

[10] Hanisch F.-G., Ragge H., Kalinski T., Meyer F., Kalbacher H., Hoffmann W. (2013). Human gastric TFF2 peptide contains an *N*-linked fucosylated *N*,*N*′-diacetyllactosediamine (LacdiNAc) oligosaccharide. Glycobiology.

[11] Hoffmann W., Jagla W. (2002). Cell type specific expression of secretory TFF peptides: Colocalization with mucins and synthesis in the brain. Int. Rev. Cytol..

[12] Hoffmann W., Jagla W., Wiede A. (2001). Molecular medicine of TFF-peptides: From gut to brain. Histol. Histopathol..

[13] Madsen J., Nielsen O., Tornoe I., Thim L., Holmskov U. (2007). Tissue localization of human trefoil factors 1, 2, and 3. J. Histochem. Cytochem..

[14] Cook G.A., Familari M., Thim L., Giraud A.S. (1999). The trefoil peptides TFF2 and TFF3 are expressed in rat lymphoid tissues and participate in the immune response. FEBS Lett..

[15] Baus-Loncar M., Kayademir T., Takaishi S., Wang T. (2005). Trefoil factor family 2 deficiency and immune response. Cell. Mol. Life Sci..

[16] Kurt-Jones E.A., Cao L., Sandor F., Rogers A.B., Whary M.T., Nambiar P.R., Cerny A., Bowen G., Yan J., Takaishi S. (2007). Trefoil family factor 2 is expressed in murine gastric and immune cells and controls both gastrointestinal inflammation and systemic immune responses. Infect. Immun..

[17] Judd L.M., Chalinor H.V., Walduck A., Pavlic D.I., Däbritz J., Dubeykovskaya Z., Wang T.C., Menheniott T.R., Giraud A.S. (2015). TFF2 deficiency exacerbates weight loss and alters immune cell and cytokine profiles in DSS colitis, and this cannot be rescued by wild-type bone marrow. Am. J. Physiol. Gastrointest. Liver Physiol..

[18] Dubeykovskaya Z., Si Y., Chen X., Worthley D.L., Renz B.W., Urbanska A.M., Hayakawa Y., Xu T., Westphalen C.B., Dubeykovskiy A. (2016). Neural innervation stimulates splenic TFF2 to arrest myeloid cell expansion and cancer. Nat. Commun..

[19] Dignass A., Lynch-Devaney K., Kindon H., Thim L., Podolsky D.K. (1994). Trefoil peptides promote epithelial migration through a transforming growth factor β-independent pathway. J. Clin. Investig..

[20] Hoffmann W. (2021). Trefoil factor family (TFF) peptides and their different roles in the mucosal innate immune defense and more: An update. Curr. Med. Chem..

[21] Otto W.R., Thim L. (2005). Trefoil factor family-interacting proteins. Cell. Mol. Life Sci..

[22] Järvå M.A., Lingford J.P., John A., Soler N.M., Scott N.E., Goddard-Borger E.D. (2020). Trefoil factors share a lectin activity that defines their role in mucus. Nat. Commun..

[23] Hoffmann W. (2015). TFF2, a MUC6-binding lectin stabilizing the gastric mucus barrier and more. Int. J. Oncol..

[24] Thim L., Mørtz E. (2000). Isolation and characterization of putative trefoil peptide receptors. Regul. Pept..

[25] Madsen J., Sorensen G.L., Nielsen O., Tornøe I., Thim L., Fenger C., Mollenhauer J., Holmskov U. (2013). A variant form of the human deleted in malignant brain tumor 1 (DMBT1) gene shows increased expression in inflammatory bowel diseases and interacts with dimeric trefoil factor 3 (TFF3). PLoS ONE.

[26] Dubeykovskaya Z., Dubeykovskiy A., Solal-Cohen J., Wang T.C. (2009). Secreted trefoil factor 2 activates the CXCR4 receptor in epithelial and lymphocytic cancer cell lines. J. Biol. Chem..

[27] Hoffmann W. (2009). Trefoil factor family (TFF) peptides and chemokine receptors: A promising relationship. J. Med. Chem..

[28] Dieckow J., Brandt W., Hattermann K., Schob S., Schulze U., Mentlein R., Ackermann P., Sel S., Paulsen F.P. (2016). CXCR4 and CXCR7 mediate TFF3-induced cell migration independently from the ERK1/2 signaling pathway. Investig. Ophthalmol. Sci..

[29] Barrera Roa G.J., Sanchez Tortolero G. (2016). Trefoil factor 3 (TFF3) from human breast milk activates PAR-2 receptors, of the intestinal epithelial cells HT-29, regulating cytokines and defensins. Bratisl. Med. J..

[30] Zhang Y., Yu G., Wang Y., Xiang Y., Gao Q., Jiang P., Zhang J., Lee W., Zhang Y. (2011). Activation of protease-activated receptor (PAR) 1 by frog trefoil factor (TFF) 2 and PAR4 by human TFF2. Cell. Mol. Life Sci..

[31] Belle N.M., Ji Y., Herbine K., Wei Y., Park J., Zullo K., Hung L.Y., Srivatsa S., Young T., Oniskey T. (2019). TFF3 interacts with LINGO2 to regulate EGFR activation for protection against colitis and gastrointestinal helminths. Nat. Commun..

[32] Zullo K., Ji Y., Wei Y., Herbine K., Maloney N., Cohen R., Pastore C., Somsouk M., Srivatsa S., Hung L.-Y. (2019). Lingo3 interacts with TFF2 to control mucosal integrity, type 1 inflammation, and colitic tissue repair. J. Immunol..

[33] Kouznetsova I., Laubinger W., Kalbacher H., Kalinski T., Meyer F., Roessner A., Hoffmann W. (2007). Biosynthesis of gastrokine-2 in the human gastric mucosa: Restricted spatial expression along the antral gland axis and differential interaction with TFF1, TFF2 and mucins. Cell. Physiol. Biochem..

[34] Albert T.K., Laubinger W., Müller S., Hanisch F.-G., Kalinski T., Meyer F., Hoffmann W. (2010). Human intestinal TFF3 forms disulfide-linked heteromers with the mucus-associated FCGBP protein and is released by hydrogen sulfide. J. Proteome Res..

[35] Stürmer R., Müller S., Hanisch F.-G., Hoffmann W. (2014). Porcine gastric TFF2 is a mucus constituent and differs from pancreatic TFF2. Cell. Physiol. Biochem..

[36] Stürmer R., Harder S., Schlüter H., Hoffmann W. (2018). Commercial porcine gastric mucin preparations, also used as artificial saliva, are a rich source for the lectin TFF2: In vitro binding studies. ChemBioChem.

[37] Heuer J., Heuer F., Stürmer R., Harder S., Schlüter H., Braga Emidio N., Muttenthaler M., Jechorek D., Meyer F., Hoffmann W. (2020). The Tumor suppressor TFF1 occurs in different forms and interacts with multiple partners in the human gastric mucus barrier: Indications for diverse protective functions. Int. J. Mol. Sci..

[38] Stürmer R., Reising J., Hoffmann W. (2019). The TFF peptides xP1 and xP4 appear in distinctive forms in the *Xenopus laevis* gastric mucosa: Indications for different protective functions. Int. J. Mol. Sci..

[39] Fra A.M., Fagioli C., Finazzi D., Sitia R., Alberini C.M. (1993). Quality control of ER synthesized proteins: An exposed thiol group as a three-way switch mediating assembly, retention and degradation. EMBO J..

[40] Reddy P., Sparvoli A., Fagioli C., Fassina G., Sitia R. (1996). Formation of reversible disulfide bonds with the protein matrix of the endoplasmic reticulum correlates with the retention of unassembled Ig light chains. EMBO J..

[41] Torres L.-F., Karam S.M., Wendling C., Chenard M.-P., Kershenobich D., Tomasetto C., Rio M.-C. (2002). Trefoil factor 1 (TFF1/pS2) deficiency activates the unfolded protein response. Mol. Med..

[42] Westley B.R., Griffin S.M., May F.E.B. (2005). Interaction between TFF1, a gastric tumor suppressor trefoil protein, and TFIZ1, a brichos domain-containing protein with homology to SP-C. Biochemistry.

[43] Reeves E.P., Ali T., Leonard P., Hearty S., O’Kennedy R., May F.E.B., Westley B.R., Josenhans C., Rust M., Suerbaum S. (2008). *Helicobacter pylori* lipopolysaccharide interacts with TFF1 in a pH-dependent manner. Gastroenterology.

[44] Braga Emidio N., Baik H., Lee D., Stürmer R., Heuer J., Elliott A.G., Blaskovich M.A., Haupenthal K., Tegtmeyer N., Hoffmann W. (2020). Chemical synthesis of human trefoil factor 1 (TFF1) and its homodimer provides novel insights into their mechanisms of action. Chem. Commun..

[45] Hanisch F.-G., Bonar D., Schloerer N., Schroten H. (2014). Human trefoil factor 2 is a lectin that binds α-GlcNAc-capped mucin glycans with antibiotic activity against *Helicobacter pylori*. J. Biol. Chem..

[46] Oinuma T., Ide S., Kawano J., Suganuma T. (1994). Purification and immunohistochemistry of *Griffonia simplicifolia* agglutinin-II-binding mucus glycoprotein in rat stomach. Glycobiology.

[47] Lang T., Klasson S., Larsson E., Johansson M.E., Hansson G.C., Samuelsson T. (2016). Searching the evolutionary origin of epithelial mucus protein components - mucins and FCGBP. Mol. Biol. Evol..

[48] Thim L., Madsen F., Poulsen S.S. (2002). Effect of trefoil factors on the viscoelastic properties of mucus gels. Eur. J. Clin. Investig..

[49] Schwarz H., Hoffmann W. (2020). Subcellular localization of the TFF peptides xP1 and xP4 in the *Xenopus laevis* gastric/esophageal mucosa: Different secretion modes reflecting diverse protective functions. Int. J. Mol. Sci..

[50] Lee H., Wang P., Hoshino H., Ito Y., Kobayashi M., Nakayama J., Seeberger P.H., Fukuda M. (2008). α1,4GlcNAc-capped mucin-type *O*-glycan inhibits cholesterol α-glucosyltransferase from *Helicobacter pylori* and suppresses *H. pylori* growth. Glycobiology.

[51] Houben T., Harder S., Schlüter H., Kalbacher H., Hoffmann W. (2019). Different forms of TFF3 in the human saliva: Heterodimerization with IgG Fc binding protein (FCGBP). Int. J. Mol. Sci..

[52] Kobayashi K., Ogata H., Morikawa M., Iijima S., Harada N., Yoshida T., Brown W.R., Inoue N., Hamada Y., Ishii H. (2002). Distribution and partial characterisation of IgG Fc binding protein in various mucin producing cells and body fluids. Gut.

[53] Li C., Wang R., Su B., Luo Y., Terhune J., Beck B., Peatman E. (2013). Evasion of mucosal defenses during *Aeromonas hydrophila* infection of channel catfish (*Ictalurus punctatus*) skin. Dev. Comp. Immunol..

[54] Wang Y., Liu Y., Liu H., Zhang Q., Song H., Tang J., Fu J., Wang X. (2017). FcGBP was upregulated by HPV infection and correlated to longer survival time of HNSCC patients. Oncotarget.

[55] Lencer W.I., Blumberg R.S. (2005). A passionate kiss, then run: Exocytosis and recycling of IgG by FcRn. Trends Cell Biol..

[56] Schwartz J.L. (2014). Fcgbp—A potential viral trap in RV144. Open AIDS J..

[57] Wong W.M., Poulsom R., Wright N.A. (1999). Trefoil peptides. Gut.

[58] Wright N.A. (1998). Aspects of the biology of regeneration and repair in the human gastrointestinal tract. Philos. Trans. R. Soc. Lond. B Biol. Sci..

[59] Rio M.-C., Chenard M.P., Wolf C., Marcellin L., Tomasetto C., Lathe R., Bellocq J.P., Chambon P. (1991). Induction of pS2 and hSP genes as markers of mucosal ulceration of the digestive tract. Gastroenterology.

[60] Wong W.M., Playford R.J., Wright N.A. (2000). Peptide gene expression in gastrointestinal mucosal ulceration: Ordered sequence or redundancy?. Gut.

[61] Viby N.-E., Nexo E., Kissow H., Andreassen H., Clementsen P., Thim L., Poulsen S.S. (2015). Trefoil factors (TFFs) are increased in bronchioalveolar lavage fluid from patients with chronic obstructive lung disease (COPD). Peptides.

[62] Hoffmann W. (2007). TFF (trefoil factor family) peptides and their potential roles for differentiation processes during airway remodeling. Curr. Med. Chem..

[63] Goldenring J.R., Nam K.T., Wang T.C., Mills J.C., Wright N.A. (2010). Spasmolytic polypeptide-expressing metaplasia and intestinal metaplasia: Time for reevaluation of metaplasias and the origins of gastric cancer. Gastroenterology.

[64] May F.E., Westley B.R. (1997). Trefoil proteins: Their role in normal and malignant cells. J. Pathol..

[65] Katoh M. (2003). Trefoil factors and human gastric cancer. Int. J. Mol. Med..

[66] Emami S., Rodrigues S., Rodrigue C.M., Le Floch N., Rivat C., Attoub S., Bruyneel E., Gespach C. (2004). Trefoil factor family (TFF) peptides and cancer progression. Peptides.

[67] Regalo G., Wright N.A., Machado J.C. (2005). Trefoil factors: From ulceration to neoplasia. Cell. Mol. Life Sci..

[68] Perry J.K., Kannan N., Grandison P.M., Mitchell M.D., Lobie P.E. (2008). Are trefoil factors oncogenic?. Trends Endocrinol. Metab..

[69] Mantovani A., Sica A., Sozziani S., Allavena P., Vecchi A., Locati M. (2004). The chemokine system in diverse forms of macrophage activation and polarization. Trends Immunol..

[70] Neurath M.F., Finotto S., Glimcher L.H. (2002). The role of Th1/Th2 polarization in mucosal immunity. Nat. Med..

[71] Sallusto F. (2016). Heterogeneity of human CD4^+^ T cells against microbes. Annu. Rev. Immunol..

[72] Ruterbusch M., Pruner K.B., Shehata L., Pepper M. (2020). In vivo CD4^+^ T cell differentiation and function: Revisiting the Th1/Th2 paradigm. Annu. Rev. Immunol..

[73] Karin M., Clevers H. (2016). Reparative inflammation takes charge of tissue regeneration. Nature.

[74] Karin M., Lawrence T., Nizet V. (2006). Innate immunity gone awry: Linking microbial infections to chronic inflammation and cancer. Cell.

[75] Grivennikov S.I., Greten F.R., Karin M. (2010). Immunity, inflammation, and cancer. Cell.

[76] Mantovani A., Allavena P., Sica A., Balkwill F. (2008). Cancer-related inflammation. Nature.

[77] Mantovani A. (2009). Cancer: Inflaming metastasis. Nature.

[78] Lin W.W., Karin M. (2007). A cytokine-mediated link between innate immunity, inflammation, and cancer. J. Clin. Investig..

[79] West N.R., McCuaig S., Franchini F., Powrie F. (2015). Emerging cytokine networks in colorectal cancer. Nat. Rev. Immunol..

[80] Baus-Loncar M., Giraud A.S. (2005). Multiple regulatory pathways for trefoil factor (TFF) genes. Cell. Mol. Life Sci..

[81] Giraud A.S., Jackson C., Menheniott T.R., Judd L.M. (2007). Differentiation of the gastric mucosa IV. role of trefoil peptides and IL-6 cytokine family signaling in gastric homeostasis. Am. J. Physiol. Gastrointest. Liver Physiol..

[82] Beck S., Sommer P., Blin N., Gött P. (1998). 5′-flanking motifs control cell-specific expression of trefoil factor genes (TFF). Int. J. Mol. Med..

[83] Van De Bovenkamp J.H., Korteland-Van Male A.M., Büller H.A., Einerhand A.W., Dekker J. (2005). Infection with *Helicobacter pylori* affects all major secretory cell populations in the human antrum. Dig. Dis. Sci..

[84] Esposito R., Morello S., Vllahu M., Eletto D., Porta A., Tosco A. (2017). Gastric TFF1 expression from acute to chronic *Helicobacter* infection. Front. Cell. Infect. Microbiol..

[85] Backert S., Naumann M. (2010). What a disorder: Proinflammatory signaling pathways induced by *Helicobacter pylori*. Trends Microbiol..

[86] Bornschein J., Malfertheiner P. (2014). *Helicobacter pylori* and gastric cancer. Dig. Dis..

[87] Dossinger V., Kayademir T., Blin N., Gött P. (2002). Down-regulation of TFF expression in gastrointestinal cell lines by cytokines and nuclear factors. Cell. Physiol. Biochem..

[88] Franic T.V., van Driel I.R., Gleeson P.A., Giraud A.S., Judd L.M. (2005). Reciprocal changes in trefoil 1 and 2 expression in stomachs of mice with gastric unit hypertrophy and inflammation. J. Pathol..

[89] Soutto M., Belkhiri A., Piazuelo M.B., Schneider B.G., Peng D., Jiang A., Washington M.K., Kokoye Y., Crowe S.E., Zaika A. (2011). Loss of TFF1 is associated with activation of NF-κB-mediated inflammation and gastric neoplasia in mice and humans. J. Clin. Investig..

[90] Guillemin K., Salama N.R., Tompkins L.S., Falkow S. (2002). Cag pathogenicity island-specific responses of gastric epithelial cells to *Helicobacter pylori* infection. Proc. Natl. Acad. Sci. USA.

[91] Tebbutt N.C., Giraud A.S., Inglese M., Jenkins B., Waring P., Clay F.J., Malki S., Alderman B.M., Grail D., Hollande F. (2002). Reciprocal regulation of gastrointestinal homeostasis by SHP2 and STAT-mediated trefoil gene activation in gp130 mutant mice. Nat. Med..

[92] Soutto M., Chen Z., Katsha A.M., Romero-Gallo J., Krishna U.S., Piazuelo M.B., Washington M.K., Peek R.M., Belkhiri A., El-Rifai W.M. (2015). Trefoil factor 1 expression suppresses *Helicobacter pylori*-induced inflammation in gastric carcinogenesis. Cancer.

[93] Wright N.A., Poulsom R., Stamp G., Van Noorden S., Sarraf C., Elia G., Ahnen D., Jeffery R., Longcroft J., Pike C. (1993). Trefoil peptide gene expression in gastrointestinal epithelial cells in inflammatory bowel disease. Gastroenterology.

[94] Ebert M.P.A., Hoffmann J., Haeckel C., Rutkowski K., Schmid R.M., Wagner M., Adler G., Schulz H.U., Roessner A., Hoffmann W. (1999). Induction of TFF1 gene expression in pancreas overexpressing transforming growth factor α. Gut.

[95] Znalesniak E.B., Fu T., Guttek K., Händel U., Reinhold D., Hoffmann W. (2016). Increased cerebral Tff1 expression in two murine models of neuroinflammation. Cell. Physiol. Biochem..

[96] Kouznetsova I., Chwieralski C.E., Balder R., Hinz M., Braun A., Krug N., Hoffmann W. (2007). Induced trefoil factor family 1 expression by trans-differentiating Clara cells in a murine asthma model. Am. J. Respir. Cell Mol. Biol..

[97] Fu T., Znalesniak E.B., Kalinski T., Möhle L., Biswas A., Salm F., Dunay I.R., Hoffmann W. (2015). TFF Peptides play a role in the immune response following oral infection of mice with *Toxoplasma gondii*. Eur. J. Microbiol. Immunol..

[98] Znalesniak E.B., Fu T., Salm F., Händel U., Hoffmann W. (2017). Transcriptional responses in the murine spleen after *Toxoplasma gondii* infection: Inflammasome and mucus-associated genes. Int. J. Mol. Sci..

[99] Koike T., Shimada T., Fujii Y., Chen G., Tabei K., Namatame T., Yamagata M., Tajima A., Yoneda M., Terano A. (2007). Up-regulation of TFF1 (pS2) expression by TNF-α in gastric epithelial cells. Gastroenterol. Hepatol..

[100] Beck S., Sommer P., dos Santos Silva E., Blin N., Gött P. (1999). Hepatocyte nuclear factor 3 (winged helix domain) activates trefoil factor gene TFF1 through a binding motif adjacent to the TATAA box. DNA Cell Biol..

[101] Hromas R., Costa R. (1995). The hepatocyte nuclear factor-3/forkhead transcription regulatory family in development, inflammation, and neoplasia. Crit. Rev. Oncol. Hematol..

[102] Zhen G., Park S.W., Nguyenvu L.T., Rodriguez M.W., Barbeau R., Paquet A.C., Erle D.J. (2007). IL-13 and epidermal growth factor receptor have critical but distinct roles in epithelial cell mucin production. Am. J. Respir. Cell Mol. Biol..

[103] Wang T.C., Goldenring J.R. (2002). Inflammation intersection: gp130 balances gut irritation and stomach cancer. Nat. Med..

[104] Judd L.M., Alderman B.M., Howlett M., Shulkes A., Dow C., Moverley J., Grail D., Jenkins B.J., Ernst M., Giraud A.S. (2004). Gastric cancer development in mice lacking the SHP2 binding site on the IL-6 family co-receptor gp130. Gastroenterology.

[105] Tomasetto C., Rio M.C. (2005). Pleiotropic effects of trefoil factor 1 deficiency. Cell. Mol. Life Sci..

[106] Hirota M., Awatsuji H., Furukawa Y., Hayashi K. (1994). Cytokine regulation of PS2 gene expression in mouse astrocytes. Biochem. Mol. Biol. Int..

[107] Ghanemi A., Yoshioka M., St-Amand J. (2020). Trefoil factor family member 2 (TFF2) as an inflammatory-induced and anti-inflammatory tissue repair factor. Animals.

[108] Royce S.G., Lim C., Muljadi R.C., Samuel C.S., Ververis K., Karagiannis T.C., Giraud A.S., Tang M.L. (2013). Trefoil factor-2 reverses airway remodeling changes in allergic airways disease. Am. J. Respir. Cell Mol. Biol..

[109] Kang W., Rathinavelu S., Samuelson L.C., Merchant J.L. (2005). Interferon gamma induction of gastric mucous neck cell hypertrophy. Lab. Investig..

[110] Shimada T., Fujii Y., Koike T., Tabei K., Namatame T., Yamagata M., Tajima A., Yoneda M., Terano A., Hraishi H. (2007). Peroxisome proliferator-activated receptor γ (PPARγ) regulates trefoil factor family 2 (TFF2) expression in gastric epithelial cells. Int. J. Biochem. Cell Biol..

[111] Nikolaidis N.M., Zimmermann N., King N.E., Mishra A., Pope S.M., Finkelman F.D., Rothenberg M.E. (2003). Trefoil factor-2 is an allergen-induced gene regulated by Th2 cytokines and STAT6 in the lung. Am. J. Respir. Cell Mol. Biol..

[112] Blanchard C., Durual S., Estienne M., Bouzakri K., Heim M.H., Blin N., Cuber J.C. (2004). IL-4 and IL-13 up-regulate intestinal trefoil factor expression: Requirement for STAT6 and de novo protein synthesis. J. Immunol..

[113] Steenwinckel V., Louahed J., Lemaire M.M., Sommereyns C., Warnier G., McKenzie A., Brombacher F., Van Snick J., Renauld J.C. (2009). IL-9 promotes IL-13-dependent paneth cell hyperplasia and up-regulation of innate immunity mediators in intestinal mucosa. J. Immunol..

[114] Yamauchi J., Kawai Y., Yamada M., Uchikawa R., Tegoshi T., Arizono N. (2006). Altered expression of goblet cell- and mucin glycosylation-related genes in the intestinal epithelium during infection with the nematode *Nippostrongylus brasiliensis* in rat. APMIS.

[115] Engevik M.A., Luk B., Chang-Graham A.L., Hall A., Herrmann B., Ruan W., Endres B.T., Shi Z., Garey K.W., Hyser J.M. (2019). *Bifidobacterium dentium* fortifies the intestinal mucus layer via autophagy and calcium signaling pathways. mBio.

[116] Manko-Prykhoda A., Allain T., Motta J.P., Cotton J.A., Feener T., Oyeyemi A., Bindra S., Vallance B.A., Wallace J.L., Beck P. (2020). *Giardia* spp. promote the production of antimicrobial peptides and attenuate disease severity induced by attaching and effacing enteropathogens via the induction of the NLRP3 inflammasome. Int. J. Parasitol..

[117] Bergstrom K.S., Guttman J.A., Rumi M., Ma C., Bouzari S., Khan M.A., Gibson D.L., Vogl A.W., Vallance B.A. (2008). Modulation of intestinal goblet cell function during infection by an attaching and effacing bacterial pathogen. Infect. Immun..

[118] Baus-Loncar M., Al-azzeh E.D., Sommer P.S., Marinovic M., Schmehl K., Kruschewski M., Blin N., Stohwasser R., Gött P., Kayademir T. (2003). Tumour necrosis factor α and nuclear factor κB inhibit transcription of human TFF3 encoding a gastrointestinal healing peptide. Gut.

[119] Baus-Loncar M., Al-azzeh E.D., Romanska H., Lalani el N., Stamp G.W., Blin N., Kayademir T. (2004). Transcriptional control of TFF3 (intestinal trefoil factor) via promoter binding sites for the nuclear factor κB and C/EBPβ. Peptides.

[120] Mashimo H., Wu D.C., Podolsky D.K., Fishman M.C. (1996). Impaired defense of intestinal mucosa in mice lacking intestinal trefoil factor. Science.

[121] Podolsky D.K., Gerken G., Eyking A., Cario E. (2009). Colitis-associated variant of TLR2 causes impaired mucosal repair because of TFF3 deficiency. Gastroenterology.

[122] Pierik M., Joossens S., Van Steen K., Van Schuerbeek N., Vlietinck R., Rutgeerts P., Vermeire S. (2006). Toll-like receptor-1, -2, and -6 polymorphisms influence disease extension in inflammatory bowel diseases. Inflamm. Bowel Dis..

[123] Kont V., Laan M., Kisand K., Merits A., Scott H.S., Peterson P. (2008). Modulation of Aire regulates the expression of tissue-restricted antigens. Mol. Immunol..

[124] Probst J.C., Skutella T., Müller-Schmid A., Jirikowski G.F., Hoffmann W. (1995). Molecular and cellular analysis of rP1.B in the rat hypothalamus: In situ hybridization and immunohisdtochemistry of a new P-domain neuropeptide. Mol. Brain Res..

[125] Hinz M., Schwegler H., Chwieralski C.E., Laube G., Linke R., Pohle W., Hoffmann W. (2004). Trefoil factor family (TFF) expression in the mouse brain and pituitary: Changes in the developing cerebellum. Peptides.

[126] Fu T., Stellmacher A., Znalesniak E.B., Dieterich D.C., Kalbacher H., Hoffmann W. (2014). Tff3 is expressed in neurons and microglial cells. Cell. Physiol. Biochem..

[127] Bernstein H.G., Dobrowolny H., Trübner K., Steiner J., Bogerts B., Hoffmann W. (2015). Differential regional and cellular distribution of TFF3 peptide in the human brain. Amino Acids.

[128] Kettenmann H., Kirchhoff F., Verkhratsky A. (2013). Microglia: New roles for the synaptic stripper. Neuron.

[129] Lefebvre O., Chenard M.P., Masson R., Linares J., Dierich A., LeMeur M., Wendling C., Tomasetto C., Chambon P., Rio M.C. (1996). Gastric mucosa abnormalities and tumorigenesis in mice lacking the pS2 trefoil protein. Science.

[130] Karam S.M., Tomasetto C., Rio M.-C. (2008). Amplification and invasiveness of epithelial progenitors during gastric carcinogenesis in trefoil factor 1 knockout mice. Cell Prolif..

[131] Hertel S.C., Chwieralski C.E., Hinz M., Rio M.-C., Tomasetto C., Hoffmann W. (2004). Profiling trefoil factor family (TFF) expression in the mouse: Identification of an antisense TFF1-related transcript in the kidney and liver. Peptides.

[132] Peterson A.J., Menheniott T.R., O’Connor L., Walduck A.K., Fox J.G., Kawakami K., Minamoto T., Ong E.K., Wang T.C., Judd L.M. (2010). *Helicobacter pylori* infection promotes methylation and silencing of trefoil factor 2, leading to gastric tumor development in mice and humans. Gastroenterology.

[133] Soutto M., Saleh M., Arredouani M.S., Piazuelo B., Belkhiri A., El-Rifai W. (2017). Loss of Tff1 promotes pro-inflammatory phenotype with increase in the levels of RORγt+ T lymphocytes and Il-17 in mouse gastric neoplasia. J. Cancer.

[134] Saukkonen K., Tomasetto C., Narko K., Rio M.-C., Ristimaki A. (2003). Cyclooxygenase-2 expression and effect of celecoxib in gastric adenomas of trefoil factor 1-deficient mice. Cancer Res..

[135] Bossenmeyer-Pourie C., Kannan R., Ribieras S., Wendling C., Stoll I., Thim L., Tomasetto C., Rio M.-C. (2002). The trefoil factor 1 participates in gastrointestinal cell differentiation by delaying G1-S phase transition and reducing apoptosis. J. Cell Biol..

[136] Buache E., Etique N., Alpy F., Stoll I., Muckensturm M., Reina-San-Martin B., Chenard M.P., Tomasetto C., Rio M.C. (2011). Deficiency in trefoil factor 1 (TFF1) increases tumorigenicity of human breast cancer cells and mammary tumor development in TFF1-knockout mice. Oncogene.

[137] Kinoshita H., Hayakawa Y., Konishi M., Hata M., Tsuboi M., Hayata Y., Hikiba Y., Ihara S., Nakagawa H., Ikenoue T. (2019). Three types of metaplasia model through *Kras* activation, *Pten* deletion, or *Cdh1* deletion in the gastric epithelium. J. Pathol..

[138] Thiem S., Eissmann M.F., Elzer J., Jonas A., Putoczki T.L., Poh A., Nguyen P., Preaudet A., Flanagan D., Vincan E. (2016). Stomach-specific activation of oncogenic KRAS and STAT3-dependent inflammation cooperatively promote gastric tumorigenesis in a preclinical model. Cancer Res..

[139] Hoffmann W. (2015). Current status on stem cells and cancers of the gastric epithelium. Int. J. Mol. Sci..

[140] Bjerknes M., Cheng H. (2002). Multipotential stem cells in adult mouse gastric epithelium. Am. J. Physiol. Gastrointest. Liver Physiol..

[141] Stange D.E., Koo B.K., Huch M., Sibbel G., Basak O., Lyubimova A., Kujala P., Bartfeld S., Koster J., Geahlen J.H. (2013). Differentiated Troy+ chief cells act as reserve stem cells to generate all lineages of the stomach epithelium. Cell.

[142] Barker N., Huch M., Kujala P., van de Wetering M., Snippert H.J., van Es J.H., Sato T., Stange D.E., Begthel H., van den Born M. (2010). Lgr5^+ve^ stem cells drive self-renewal in the stomach and build long-lived gastric units in vitro. Cell Stem Cell.

[143] Hayakawa Y., Jin G., Wang H., Chen X., Westphalen C.B., Asfaha S., Renz B.W., Ariyama H., Dubeykovskaya Z.A., Takemoto Y. (2015). CCK2R identifies and regulates gastric antral stem cell states and carcinogenesis. Gut.

[144] Shishkin S.S., Eremina L.S., Kovalev L.I., Kovaleva M.A. (2013). AGR2, ERp57/GRP58, and some other human protein disulfide isomerases. Biochemistry.

[145] Hauri H.-P., Appenzeller C., Kuhn F., Nufer O. (2000). Lectins and traffic in the secretory pathway. FEBS Lett..

[146] Karasawa F., Shiota A., Goso Y., Kobayashi M., Sato Y., Masumoto J., Fujiwara M., Yokosawa S., Muraki T., Miyagawa S. (2012). Essential role of gastric gland mucin in preventing gastric cancer in mice. J. Clin. Investig..

[147] Soutto M., Chen Z., Bhat A.A., Wang L., Zhu S., Gomaa A., Bates A., Bhat N.S., Peng D., Belkhiri A. (2019). Activation of STAT3 signaling is mediated by TFF1 silencing in gastric neoplasia. Nat. Commun..

[148] Farrell J.J., Taupin D., Koh T.J., Chen D., Zhao C.M., Podolsky D.K., Wang T.C. (2002). TFF2/SP-deficient mice show decreased gastric proliferation, increased acid secretion, and increased susceptibility to NSAID injury. J. Clin. Investig..

[149] Xue L., Aihara E., Podolsky D.K., Wang T.C., Montrose M.H. (2010). In vivo action of trefoil factor 2 (TFF2) to speed gastric repair is independent of cyclooxygenase. Gut.

[150] Fox J.G., Rogers A.B., Whary M.T., Ge Z., Ohtani M., Jones E.K., Wang T.C. (2007). Accelerated progression of gastritis to dysplasia in the pyloric antrum of TFF2^−/−^ C57BL6 x Sv129 *Helicobacter pylori*-infected mice. Am. J. Pathol..

[151] Soriano-Izquierdo A., Gironella M., Massaguer A., May F.E., Salas A., Sans M., Poulsom R., Thim L., Piqué J.M., Panés J. (2004). Trefoil peptide TFF2 treatment reduces VCAM-1 expression and leukocyte recruitment in experimental intestinal inflammation. J. Leukoc. Biol..

[152] Vandenbroucke K., Hans W., Van Huysse J., Neirynck S., Demetter P., Remaut E., Rottiers P., Steidler L. (2004). Active delivery of trefoil factors by genetically modified *Lactococcus lactis* prevents and heals acute colitis in mice. Gastroenterology.

[153] McBerry C., Egan C.E., Rani R., Yang Y., Wu D., Boespflug N., Boon L., Butcher B., Mirpuri J., Hogan S.P. (2012). Trefoil factor 2 negatively regulates type 1 immunity against *Toxoplasma gondii*. J. Immunol..

[154] Shah A.A., Mihalj M., Ratkay I., Lubka-Pathak M., Balogh P., Klingel K., Bohn E., Blin N., Baus-Lončar M. (2012). Increased susceptibility to *Yersinia enterocolitica* Infection of Tff2 deficient mice. Cell. Physiol. Biochem..

[155] McCarthy A.J., Birchenough G.M.H., Taylor P.W. (2019). Loss of trefoil factor 2 sensitizes rat pups to systemic infection with the neonatal pathogen *Escherichia coli* K1. Infect. Immun..

[156] Birchenough G.M., Johansson M.E., Stabler R.A., Dalgakiran F., Hansson G.C., Wren B.W., Luzio J.P., Taylor P.W. (2013). Altered innate defenses in the neonatal gastrointestinal tract in response to colonization by neuropathogenic *Escherichia coli*. Infect. Immun..

[157] Hung L.-Y., Sen D., Oniskey T.K., Katzen J., Cohen N.A., Vaughan A.E., Nieves W., Urisman A., Beers M.F., Krummel M.F. (2019). Macrophages promote epithelial proliferation following infectious and non-infectious lung injury through a Trefoil factor 2-dependent mechanism. Mucosal Immunol..

[158] Wills-Karp M., Rani R., Dienger K., Lewkowich I., Fox J.G., Perkins C., Lewis L., Finkelman F.D., Smith D.E., Bryce P.J. (2012). Trefoil factor 2 rapidly induces interleukin 33 to promote type 2 immunity during allergic asthma and hookworm infection. J. Exp. Med..

[159] Buzzelli J.N., Chalinor H.V., Pavlic D.I., Sutton P., Menheniott T.R., Giraud A.S., Judd L.M. (2015). IL33 is a stomach alarmin that initiates a skewed Th2 response to injury and infection. Cell. Mol. Gastroenterol. Hepatol..

[160] Vermeer P.D., Einwalter L.A., Moninger T.O., Rokhlina T., Kern J.A., Zabner J., Welsh M.J. (2003). Segregation of receptor and ligand regulates activation of epithelial growth factor receptor. Nature.

[161] Katz-Kiriakos E., Steinberg D.F., Kluender C.E., Osorio O.A., Newsom-Stewart C., Baronia A., Byers D.E., Holtzman M.J., Katafiasz D., Bailey K.L. (2021). Epithelial IL-33 appropriates exosome trafficking for secretion in chronic airway disease. JCI Insight.

[162] Engevik K.A., Hanyu H., Matthis A.L., Zhang T., Frey M.R., Oshima Y., Aihara E., Montrose M.H. (2019). Trefoil factor 2 activation of CXCR4 requires calcium mobilization to drive epithelial repair in gastric organoids. J. Physiol..

[163] Händel T.M., Dyer D.P. (2021). Perspectives on the biological role of chemokine:glycosaminoglycan interactions. J. Histochem. Cytochem..

[164] Park S.-W., Zhen G., Verhaeghe C., Nakagami Y., Nguyenvu L.T., Barczak A.J., Killeen N., Erle D.J. (2009). The protein disulfide isomerase AGR2 is essential for production of intestinal mucus. Proc. Natl. Acad. Sci. USA.

[165] Johansson M.E., Larsson J.M., Hansson G.C. (2011). The two mucus layers of colon are organized by the MUC2 mucin, whereas the outer layer is a legislator of host-microbial interactions. Proc. Natl. Acad. Sci. USA.

[166] Johansson M.E., Hansson G.C. (2016). Immunological aspects of intestinal mucus and mucins. Nat. Rev. Immunol..

[167] Johansson M.E., Gustafsson J.K., Sjöberg K.E., Petersson J., Holm L., Sjövall H., Hansson G.C. (2010). Bacteria penetrate the inner mucus layer before inflammation in the dextran sulfate colitis model. PLoS ONE.

[168] Hoebler C., Gaudier E., De Coppet P., Rival M., Cherbut C. (2006). MUC genes are differently expressed during onset and maintenance of inflammation in dextran sodium sulfate-treated mice. Dig. Dis. Sci..

[169] Kjellev S., Thim L., Pyke C., Poulsen S.S. (2007). Cellular localization, binding sites, and pharmacologic effects of TFF3 in experimental colitis in mice. Dig. Dis. Sci..

[170] Madsen J., Mollenhauer J., Holmskov U. (2010). Review: Gp-340/DMBT1 in mucosal innate immunity. Innate Immun..

[171] Arnold P., Rickert U., Helmers A.K., Spreu J., Schneppenheim J., Lucius R. (2016). Trefoil factor 3 shows anti-inflammatory effects on activated microglia. Cell Tissue Res..

[172] Leclaire C., Lecointe K., Gunning P.A., Tribolo S., Kavanaugh D.W., Wittmann A., Latousakis D., MacKenzie D.A., Kawasaki N., Juge N. (2018). Molecular basis for intestinal mucin recognition by galectin-3 and C-type lectins. FASEB J..

[173] García Caballero G., Kaltner H., Kutzner T.J., Ludwig A.K., Manning J.C., Schmidt S., Sinowatz F., Gabius H.J. (2020). How galectins have become multifunctional proteins. Histol. Histopathol..

[174] Wesener D.A., Dugan A., Kiessling L.L. (2017). Recognition of microbial glycans by soluble human lectins. Curr. Opin. Struct. Biol..

[175] Meanwatthana J., Majam T. (2021). Interleukin-6 antagonists: Lessons from cytokine release syndrome to the therapeutic application in severe COVID-19 infection. J. Pharm. Pract..

[176] Omar O.M., Suotto M., Bhat N.S., Bhat A.A., Lu H., Chen Z., El-Rifai W. (2018). TFF1 antagonizes TIMP-1 mediated proliferative functions in gastric cancer. Mol. Carcinogen..

[177] Rabinovich G.A., Rubinstein N., Toscano M.A. (2002). Role of galectins in inflammatory and immunomodulatory processes. Biochim. Biophys. Acta.

[178] Johannssen T., Lepenies B. (2017). Glycan-Based Cell Targeting To Modulate Immune Responses. Trends Biotechnol..

[179] Sacchettini J.C., Baum L.G., Brewer C.F. (2001). Multivalent protein-carbohydrate interactions. a new paradigm for supermolecular assembly and signal transduction. Biochemistry.

[180] Rabinovich G.A., Toscano M.A., Jackson S.S., Vasta G.R. (2007). Functions of cell surface galectin-glycoprotein lattices. Curr. Opin. Struct. Biol..

[181] Patel U., Rajasingh S., Samanta S., Cao T., Dawn B., Rajasingh J. (2017). Macrophage polarization in response to epigenetic modifiers during infection and inflammation. Drug Discov. Today.

[182] Bulitta C.J., Fleming J.V., Raychowdhury R., Taupin D., Rosenberg I., Wang T.C. (2002). Autoinduction of the trefoil factor 2 (TFF2) promoter requires an upstream *cis*-acting element. Biochem. Biophys. Res. Commun..

[183] Martin V., Ribieras S., Rio M.-C., Dante R. (1998). The estrogen responsive element of the *pS2* gene is recognized by a methylation sensitive DNA binding protein. Biol. Chem..

[184] Ribieras S., Lefèbvre O., Tomasetto C., Rio M.-C. (2001). Mouse *Trefoil Factor* genes: Genomic organization, sequences and methylation analyses. Gene.

[185] Carvalho R., Kayademir T., Soares P., Canedo P., Sousa S., Oliveira C., Leistenschneider P., Seruca R., Gött P., Blin N. (2002). Loss of heterozygosity and promoter methylation, but not mutation, may underlie loss of TFF1 in gastric carcinoma. Lab. Investig..

[186] Braga Emidio N., Hoffmann W., Brierley S.M., Muttenthaler M. (2019). Trefoil factor family: Unresolved questions and clinical perspectives. Trends Biochem. Sci..

[187] Royce S.G., Lim C., Muljadi R.C., Tang M.L.K. (2011). Trefoil factor 2 regulates airway remodeling in animal models of asthma. J. Asthma.

[188] Popp J., Schicht M., Garreis F., Klinger P., Gelse K., Sesselmann S., Tsokos M., Etzold S., Stiller D., Claassen H. (2019). Human synovia contains trefoil factor family (TFF) peptides 1-3 although synovial membrane only produces TFF3: Implications in osteoarthritis and rheumatoid arthritis. Int. J. Mol. Sci..

[189] Paterson R.W., Bartlett J.W., Blennow K., Fox N.C., Shaw L.M., Trojanowski J.Q., Zetterberg H., Schott J.M. (2014). Cerebrospinal fluid markers including trefoil factor 3 are associated with neurodegeneration in amyloid-positive individuals. Transl. Psychiatry.

